# The bone metastasis niche in breast cancer-potential overlap with the haematopoietic stem cell niche *in vivo*

**DOI:** 10.1016/j.jbo.2019.100244

**Published:** 2019-06-07

**Authors:** Gloria Allocca, Russell Hughes, Ning Wang, Hannah K Brown, Penelope D Ottewell, Nicola J Brown, Ingunn Holen

**Affiliations:** Department of Oncology and Metabolism, Medical School, University of Sheffield, UK

**Keywords:** Bone metastasis, Animal models, Breast cancer, Hematopoietic stem cell, ANOVA, Analysis of variance, CTC, Circulating tumour cell, DAPI, 4′,6-diamidino-2-phenylindole, DTC, Disseminated tumour cell, EDTA, Ethylenediaminetetraacetic acid, ER, Estrogen Receptor, FBS, Foetal bovine serum, GFP, Green fluorescent protein, HSC, Hematopoietic stem cell, IC, Intra cardiac, IV, Intra venous, Luc2, Luciferase2, OVX, Ovariectomy, ROI, Region of interest, TSP-1, thrombospondin-1, µCT, Microcomputed tomography

## Abstract

Background: Bone metastasis is one of the most common complications of advanced breast cancer. During dissemination to bone, breast cancer cells locate in a putative ‘metastatic niche’, a microenvironment that regulates the colonisation, maintenance of tumour cell dormancy and subsequent tumour growth. The precise location and composition of the bone metastatic niche is not clearly defined. We have used *in vivo* models of early breast cancer dissemination to provide novel evidence that demonstrates overlap between endosteal, perivascular, HSC and the metastatic niche in bone.

Methods: Estrogen Receptor (ER) +ve and -ve breast cancer cells were labelled with membrane dyes Vybrant-DiD and Vybrant-CM-DiI and injected via different routes in BALBc/nude mice of different ages. Two-photon microscopy was used to detect and quantitate tumour cells and map their location within the bone microenvironment as well as their distance to the nearest bone surface compared to the nearest other tumour cell. To investigate whether the metastatic niche overlapped with the HSC niche, animals were pre-treated with the CXCR4 antagonist AMD3100 to mobilise hematopoietic (HSCs) prior to injection of breast cancer cells.

Results: Breast cancer cells displayed a characteristic pattern of homing in the long bones, with the majority of tumour cells seeded in the trabecular regions, regardless of the route of injection, cell-line characteristics (ER status) or animal age. Breast cancer cells located in close proximity to the nearest bone surface and the average distance between individual tumour cells was higher than their distance to bone. Mobilisation of HSCs from the niche to the circulation prior to injection of cell lines resulted in increased numbers of tumour cells disseminated in trabecular regions.

Conclusion: Our data provide evidence that homing of breast cancer cells is independent of their ER status and that the breast cancer bone metastasis niche is located within the trabecular region of bone, an area rich in osteoblasts and microvessels. The increased number of breast cancer cells homing to bone after mobilisation of HSCs suggests that the HSC and the bone metastasis niche overlap.

## 1 Introduction

Metastatic breast cancer remains a major cause of female cancer-related deaths, despite the improvements in early detection and therapy introduced over the past decades [1], [2]. The most frequent metastatic site is the skeleton, but other distant organs including brain, lungs and liver are also affected [3]. Secondary disease, particularly skeletal metastasis, can develop decades after the detection and treatment of the primary tumour. Recent studies show that the risk of distant recurrence increases at a steady rate for at least 20 years [4]. Bone metastases are associated with major complications such as hypercalcaemia, pathological fractures and spinal cord compression, resulting in a considerable reduction in quality of life [5]. Although the focus of intensive research, the mechanisms initiating the metastatic process are not fully understood [6], and the lack of available patient bone biopsies containing tumour cells hampers the investigation of bone metastasis in humans. Therefore, pre-clinical models are extensively used to investigate the mechanisms underpinning metastatic spread, including to the skeleton, in order to identify potential therapeutic targets.

The development of metastasis in distant organs is a multistep process that begins with the detachment of cancer cells from the primary tumour, via their invasion of the extra-cellular matrix, entry into the blood stream and the eventual colonisation and outgrowth at secondary sites [3], [7], [8]. However, only a small proportion of the cells that escape from the primary tumour will successfully colonise a distant organ, with the majority of circulating tumour cells (CTCs) being recognised and eliminated by the immune system, undergoing spontaneous apoptosis or re-entering the blood stream and potentially colonising other organ(s) [9], [10], [11], [12]. Once the CTCs have colonised the bone microenvironment, they are referred to as disseminated tumour cells (DTCs), for which there are several different fates: (1) death by spontaneous apoptosis or elimination by the host immune system; (2) return to the circulation; (3) entry into a quiescent or dormant state; and (4) proliferation with induction of cancer-induced bone disease [7], [9], [10], [11], [12]. It is hypothesised that DTCs, particular those derived from prostate and breast cancers, colonise specialized regions of bone microenvironment in which they can be held in a quiescent state; ‘The Metastatic Niche(s)’ [3]. This bone region is characterized by a complex milieu of cytokines, hormones and soluble factors produced by the different components of the surrounding niches, creating a rich soil for seeding of the tumour cells. This metastatic niche strongly influences the fate of DTCs arriving in bone, driving them towards either dormancy or proliferation and development of overt bone metastases [13]. The precise position and composition of the metastatic niche has not yet been established, but studies from murine model systems have provided evidence that tumour cells locate in regions of bone where the HSC niche, endosteal niche and perivascular niche overlap [14], [15], [16], [17]. Cells of the osteoblastic lineage are reported to be involved in the tumour establishment and progression of bone metastases in pre-clinical models of both breast and prostate cancer [18], [19]. In addition, Shiozawa et al. elegantly showed how prostate cancer cells and HSCs locate within the same niche [20], [21], supporting the hypothesis of an overlap between niches in bone. The HSC niche is found in close proximity to the endosteal surface of the trabecular bone, an area that is highly vascularised [17], [22], [23], [24], [25]. Whilst the contribution of the vasculature in the early stage of bone metastasis is not fully elucidated, a recent study has provided some evidence that the perivascular niche is a key regulator of breast cancer cell dormancy. Ghajar et al. used a range of *in vitro* model systems to demonstrate that breast cancer cells associated with a quiescent microvasculature promotes tumour cell dormancy, whereas this is reversed during vessel sprouting [26]. It is a well-established concept that the microenvironment plays an important role in all the stages of bone metastasis, however the precise cellular composition of the metastatic niche remains to be defined.

To date, most studies of bone metastasis have focused on advanced stages of cancer-induced disease, where the micro- or macro-metastases are already established. In contrast, much less information is available regarding the early stages of breast cancer bone colonisation, when tumour cells remain in a dormant state within the bone marrow. There are several key questions that remain to be addressed. Do cancer cells compete with each other, or with other bone-residing cell populations, for access to a limited number of suitable niches that could become saturated? We also do not know the fate of tumour cells that arrive in bone to find that the prime niches are occupied. The application of novel technologies, such as two-photon microscopy and the use of lipophilic dyes that are retained in non-proliferating (tumour) cells, has facilitated studies of the initial stages of the metastatic process in model systems, as well as how changes in bone cell populations affects the homing of tumour cells [27], [28]. We have combined these approaches to quantify and map single breast cancer cells of different molecular subtypes within the long bones of mice, determining their precise position in relation to the calcified structures, the neighbouring tumour cells and their spatial relationship to key components of the bone microenvironment. We demonstrate that ER+ve and ER-ve breast cancer cells display the same homing pattern in bone and that this is independent of the injection route used or the age of the animal. Finally, we provide novel evidence that breast cancer cells home to the HSC niche.

## 2 Materials and methods

### 2.1 Tumour cell lines

MDA-MB-231-GFP-IV [29], T47D and MCF7 breast cancer cell lines (ATCC) were cultured in RPMI 1640 supplemented with 10% FBS (Life Technologies, Paisley, UK /Invitrogen) at 37 °C 5%CO_2,_ MDA-MB-231-NW1-Luc2 cells were cultured in DMEM (Life Technologies, Paisley, UK) + Pyruvate medium enriched with 100 U/mL penicillin/streptomycin and 10% FBS (Sigma Aldrich Co Ltd, Poole, UK). Prior to the injections, tumour cells were labelled either with the lipophilic membrane dye Vybrant-CM-DiI or Vybrant-DiD (Life Technologies Ltd, Paisley, UK) according to the manufacturer's instructions.

### 2.2 Animal models

Six-week old and twelve-week old female BALB/c nude mice (Charles River, UK) were used to assess the homing of breast cancer cells in bone and to establish any effects of modification of the niche. Mice were housed in a controlled environment with a 12 h light/dark cycle at 22 °C. They were provided with *ad libitum* access to *2018 Teklad* Global 18% protein rodent diet containing 1.01% Calcium (Harlan Laboratories, UK) and water, and housed in groups of 5/6 in ventilated cages. The Research Ethics Committee for animal experimentation, of the University of Sheffield, UK reviewed and the Home Office approved all the work included in this manuscript. All *in vivo* experiments were performed in accordance with the UK Animals (Scientific Procedures) Act 1986 Home Office regulations under the authority of PPL 70/8964 and PPL 70/8799.

### 2.3 Bone homing studies

To assess the homing of breast cancer tumour cells to bone, 12-week old female BALB/c nude mice were injected intravenously (i.v.) with 1 × 10^5^ MDA-MB-231-GFP-IV breast cancer cells labelled either with the membrane dye Vybrant-CM-DiI or Vybrant-DiD and culled on day 5. Hind limbs were dissected, muscles removed, femora and tibiae separated and processed for imaging as described below.

To investigate whether different routes of administration affected the pattern of homing of breast cancer cells, 12-week old female BALB/c nude mice were injected either intravenously (*n* = 1) or via the intracardiac (i.c) route (*n* = 5) with 1 × 10^5^ MDA-MB-231-GFP-IV breast cancer cells labelled with the membrane dye Vybrant-DiD and then culled on day 5 and long bones collected.

To establish whether the oestrogen receptor (ER) status of the breast cancer cells affected their pattern of bone homing, 1 × 10^5^ Vybrant-DiD labelled ER-ve MDA-MB-231-GFP-IV cells, ER+ve MCF-7 or T47D cells were injected i.c. in 12-week old female BALB/c nude mice (*n* = 5/group). Long bones were collected 5 days after cell injection and processed for imaging as described below.

### 2.4 Modification of the HSC niche

To confirm that AMD3100 mobilises HSC/progenitor cells (PCs) from bone marrow niches into the circulation, 12-week old female BALB/c-Nude mice were treated with AMD3100 (5 mg/kg, i.p.) or Saline daily for 5 days. Three hours after the last drug administration, mice were culled and peripheral blood isolated by cardiac puncture using syringes containing 4% sodium citrate. Blood was centrifuged to remove the serum and red blood cells lysed with ammonium chloride solution (BioLegend Ltd, UK). Peripheral blood mononuclear cells were resuspended in IMDM supplemented with 2% FCS and 100 u/mL Penicillin/Streptomycin at a density of 2 × 10^6^ cells/mL. Cell suspensions were diluted 1:10 with MethoCult GF 3434 medium (Stem Cell Technologies), seeded into 6-well plates and maintained in an humidified atmosphere at 37 °C with 5% CO_2_ for 11 days to allow colony formation. Bright field images of colonies were taken using a Leica DMI4000B microscope.

To assess whether the mobilisation of hematopoietic stem cells (HSCs) would affect homing of breast cancer cells in bone, 12-week old female BALB/c nude were injected i.p. with the CXCR4 antagonist AMD3100 (Sigma-Aldrich) 5 mg/kg (100 µl) or PBS daily for 5 days. 24 h after the last injection, animals were injected i.v with 1 × 10^5^ MDA-MB-231-GFP-IV Vybrant-DiD -labelled cells and culled on day 10.

### 2.5 Comparison between young and mature animals

To compare the seeding of breast cancer cells in the bone microenvironment of young and mature animals 6- and 12-week old female BALB/c nude mice (*n* = 8/group) were injected i.v. with 1 × 10^5^ MDA-MB-231-GFP-IV cells labelled with the membrane dye Vybrant-CM-DiI. Animals were culled 5 days after tumour cell injection.

### 2.6 µCT analysis

Microcomputed tomography (µCT) analysis was carried out on the resected long bones *ex vivo* using a Skyscan 1272 X-ray-computed microtomograph (Skyscan). Image acquisition was performed using a voltage of 50 kV, a current of 200 µA, a medium camera resolution of 2016 × 1344, an aluminium filter of 0.5 mm and a pixel size of 4.3 µm. Images were captured of proximal tibias every 0.7° through a 180° rotation. Acquired images were reconstructed and analyzed using NRecon and CTAn software (Skyscan).

### 2.7 Two-photon microscopy

Animals were culled, hind limbs dissected, muscles removed, femora and tibiae separated and collected snap frozen in liquid nitrogen and stored at −80 °C. Cryopreserved long bones were then embedded into Cryo-M-Bed (Instrument Co. Ltd, Huntingdon, UK) and the bone marrow exposed using a Bright OTF Cryostat and a 3020 microtome (Bright Instrument Co. Ltd, Huntingdon, UK). A stack area of exposed bone marrow (2104 µm × 2525 µm 70 µm depth) was captured using a Zeiss LSM510 NLO upright 2-photon/confocal microscope (Carl Zeiss Inl, Cambridge, UK). DiD-labelled and CM-Dil-labelled breast cancer cells were visualised using a 633 nm HeNe and 543 HeNe lasers, respectively. The bone structure was detected with a Chameleon 2-photon laser at 900 nm (Coherent, Santa Clara, CA.). The Volocity 3D Image Analysis software 6.01 (PerkinElmer, Cambridge, UK) was used to quantify the abundance of Vybrant-CM-DiI or Vybrant-DiD events, their distance to the nearest bone surface and to other DTCs [27]. The quantification of dye-labelled tumour cells and their spatial distribution were performed in two different regions of interest (ROIs, Fig. 2B) from which the cortical bone was excluded.

### 2.8 Confocal microscopy

Long bones were fixed in 4%PFA, decalcified in 0.5 M EDTA, embedded in gelatin and frozen at −80 °C [27]. Immunofluorescence was performed on 30 µm thick cryosections (three non-consecutive levels) for each bone. Tissue sections were permeabilized for 30 min in 0.3% Triton X-100, where appropriate Streptavidin/Biotin blocking (Vector Labs) was performed. Cryosections were incubated for 1 h with primary antibody against murine Endomucin (2 µg/mL, Santa Cruz Biotechnology), Osteopontin (R&D Systems) or human CD29 and CD59 (10 µg/mL, BioLegend). Tissue sections were washed with PBS and incubated with either a fluorescently-conjugated (5 µg/mL) or biotinylated secondary (7.5 µg/mL) antibody followed by fluorescently-conjugated streptavidin. Tissue sections were washed with PBS and incubated with either a fluorescently-conjugated or biotinylated secondary antibody followed by fluorescently-conjugated streptavidin. All incubations were carried out at ambient temperature and ProLong Gold Antifade reagent (Thermo Fisher) to mount the glass coverslips. Images of the immunofluorescence staining were captured with Nikon A1 (Nikon Instruments Europe) or Zeiss LSM 880 AiryScan (Carl Zeiss Microscopy GmBH) confocal systems.

### 2.9 Statistical analysis

Statistical analyses were performed using GraphPad Prism software (Version 6.0 and 7.0). Student T-tests and Two-way ANOVA and Tukey's *post hoc* test were used as indicated in each figure legend. A *p*-value of *p* < 0.05 was considered significant.

## 3 Results

**Fig. 1:**
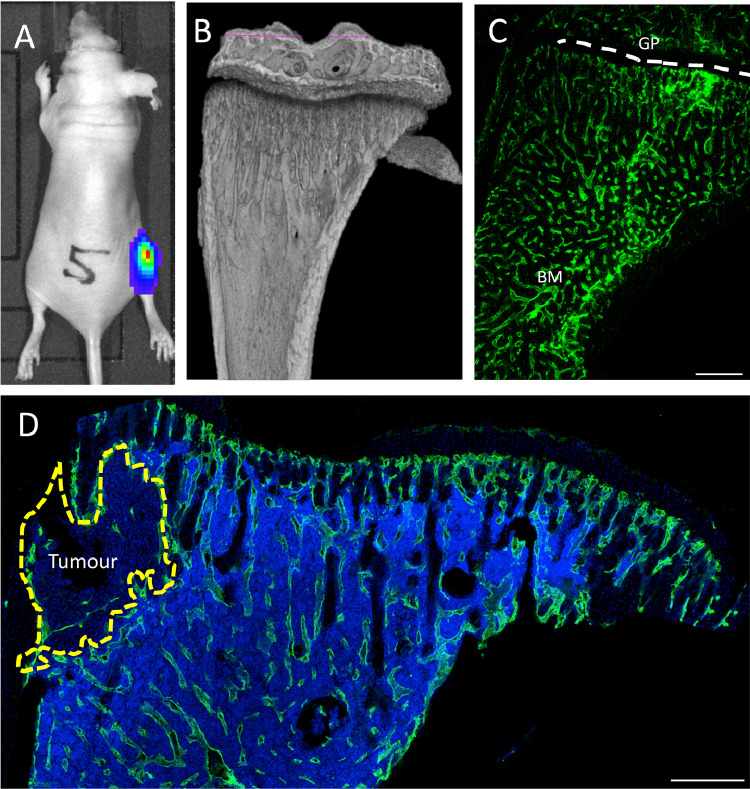
*In vivo* tumour model and location of the bone metastatic niche. (A) Representative image of the breast cancer bone metastasis animal model. Female BALB/c nude mice were injected i.c. MDA-MB-231-GFP cells transfected with luciferase, tumour growth was detected in the hind limbs using an *in vivo* imaging system (IVIS). (B) Representative micro-computed tomography reconstruction illustrating the trabecular architecture of the tibia. (C) Immunofluorescence staining using antibody against the endothelial marker endomucin to visualise the extensive microvascular network of long bones. (D) Immunofluorescence staining of a tumour bearing tibial section using an antibody specific for the endothelial cell marker endomucin. Dotted yellow line = tumour outline, BM = bone marrow, GP = growth plate, green = Endomucin and blue = DAPI, scale bar = 200um. (For interpretation of the references to color in this figure legend, the reader is referred to the web version of this article.)

During the metastatic process, breast cancer cells home to a putative bone metastatic niche. In our *in vivo* models of bone metastasis, intra-cardiac injection of Luc2 expressing breast cancer cells results in the majority of tumours developing in the highly vascularised metaphysis region of the long bones (Fig. 1). To define the location of the metastatic niche in our model, we therefore analysed the tibiae and femora to assess the location of breast cancer cells during the early stages (<10 days after tumour cell injection as defined by our previously published studies of bone colonisation [30].

### 3.1 MDA-MB-231-GFP-IV bone seeking cells preferentially home to the trabecular region of bone

In previous studies we have demonstrated that the bone seeking MDA-MB-231-IV cells form detectable tumours in long bones two weeks after injection into the tail vein of 6-week old animals, in which tumour growth is supported by active bone turnover [29]. The aim of the current study was to investigate tumour cell dissemination in the *absence* of growing colonies. We therefore used 12-week old animals, in which we have shown that low levels of bone turnover supports DTC dormancy rather than outgrowth [31]. Animals were culled 5 days after tumour cell injection, allowing DTCs to home to bone, but without starting to proliferate resulting in loss of the fluorescent membrane label. Moreover, at this time point, DTCs that have not reach a supportive niche will have undergone apoptosis and been cleared from the bone marrow, allowing quantification of the DTCs in bone that contain the metastasis-initiating population.

**Fig. 2:**
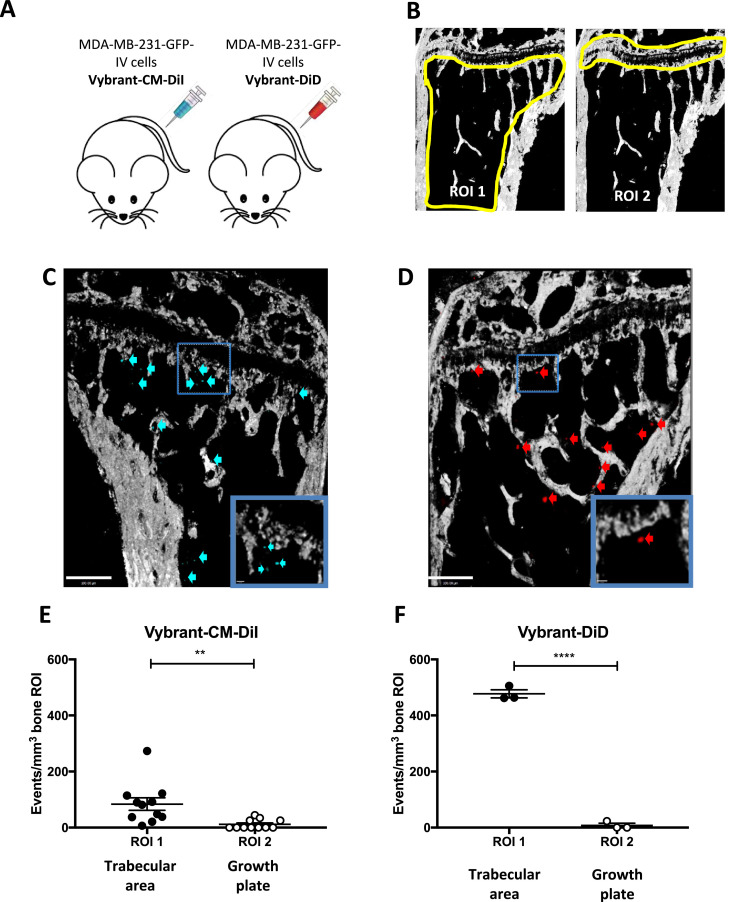
Homing of the bone-seeking MDA-MB-231-GFP-IV cell-line. (A) Experimental outline of the *in vivo* study. 12-week old female BALB/c nude mice were injected intravenously (i.v.) with PBS on day 0 followed by 1 × 10^5^ MDA-MB-231-GFP-IV cells labelled with Vybrant-CM-DiI (*n* = 6) or Vybrant-DiI (*n* = 2) on day 7. Animals were culled on day 12 and tibiae and femora were collected for analysis by two-photon microscopy. (B) Schematic representation of the regions of interest (ROIs) of two-photon bone scan analysed: ROI1 consists of the trabecular bone region and ROI2 of the area of bone immediately adjacent to/including the growth plate. Cortical bone was excluded. (C) and (D) are examples of two-photon scan showing bone (white), Vybrant-CM-DiI^+^cells (blue and blue arrows in panel C) and Vybrant-DID^+^cells (red and red arrows in panel D). Scale bars 100 µm.(E) and (F) Show the number of Vybrant-CM-DiI^+^ events/mm^3^ (*n* = 11 bones analysed), Vybrant-DiD^+^(*n* = 3 bones analysed) events. **p ≤ 0.005 and ****p < 0.0001 student's *t*-test, graphs show mean ± SEM. (For interpretation of the references to color in this figure legend, the reader is referred to the web version of this article.)

To investigate whether breast cancer cells have a preferential pattern of homing within the long bones, 12-week old mice were injected with bone-seeking MDA-MB-231-IV breast cancer cells labelled with either Vybrant-CM-DiI (Fig. 2C, E) or Vybrant-DID (Fig. 2D, F) and the number of events located in two different regions of interested (ROIs) were quantified by two-photon microscopy. The regions of interest encompassed either the trabecular bone (ROI1) and the area immediately adjacent to the growth plate (ROI2), one fluorescent event detected is equivalent to one tumour cell (Fig. 2B).

MDA-MB-231-GFP-IV cells localised preferentially in the trabecular region of the bone marrow (ROI1) rather than in the growth plate area (ROI2). Irrespective of the dye used to label the cells, the events/mm^3^ recorded in ROI1 were significantly higher compared to those in ROI2 (Vybrant-CM-DiI labelled cells *p* = 0.0047 and Vybrant-DiD labelled cells *p* < 0.0001) (Fig. 2E and F). Although the overall number of with Vybrant-CM-DiI^+^ events was lower when compared to Vybrant-DiD^+^ events, the homing pattern was comparable irrespective of the label used. These results demonstrate that in this *in vivo* model of low bone turnover, disseminated breast cancer cells mainly locate to the trabecular region of the metaphysis.

### 3.2 MDA-MB-231-GFP-IV cells locate close to the bone surface

**Fig. 3:**
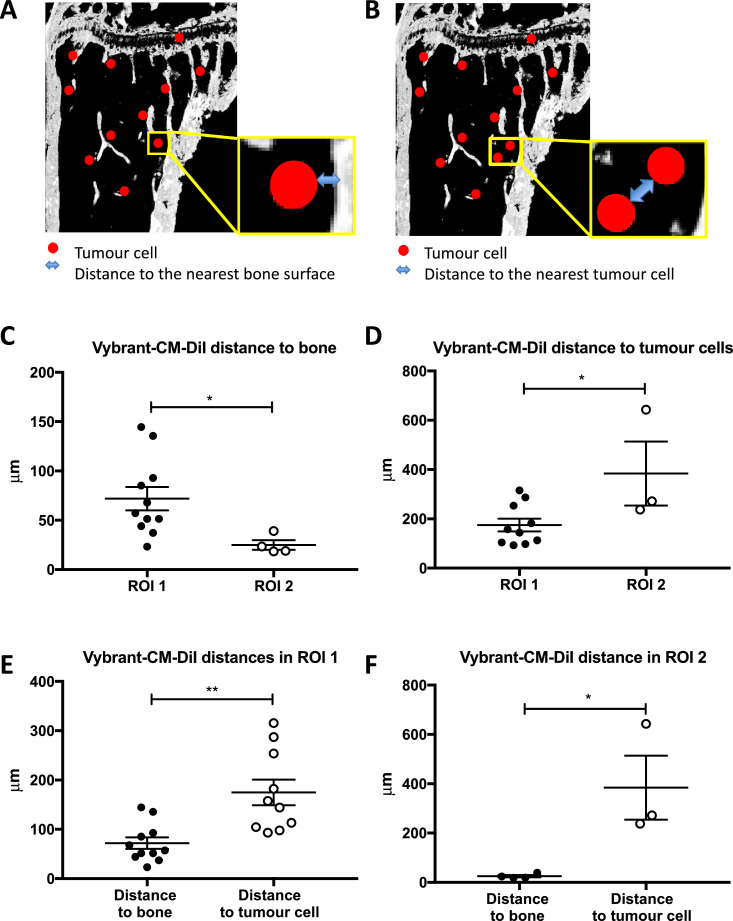
Location of MDA-MB-231-GFP-IV cells in the bone microenvironment. (A) Schematic illustration of the distances measured from the edge of the tumour cells to the nearest bone surface and (B) to the closest other cancer cell. Graphs show the distance of Vybrant-CM-DiI cells to the bone surface (C) and to other tumour cells (D) (*n* = 11 bones analysed). The comparison between the distances to the bone surface and to other tumour cells in ROI1 is shown in (E) and in ROI2 in (F). *p ≤ 0.05 and **p ≤ 0.01, student's *t*-test, graphs show mean ± SEM.

Tumour cells interact with the various components of the bone metastatic niche to develop into overt metastases, in particular with the bone-forming (osteoblasts) and the bone-resorbing (osteoclasts) cells [32]. In order to determine the precise location of MDA-MB-231-IV cells within the microenvironment, we measured the distance from the edge of each tumour cell to the nearest bone surface (covered with osteoblasts). In order to determine whether DTCs tended to co-locate in the same niche and/or form small clusters, the distance to the closest other DTC was also measured. The distances were obtained using Volocity 3D Image Analysis software as illustrated in Fig. 3A and B. In ROI1, the mean distance between Vybrant-CM-Dil labelled tumour cells and the nearest bone surface was 71.90 ± 11.82 µm (range 23–144 µm), for Vybrant-DiD cells was 108.27 ± 42.19µm (63–192 µm) whilst the overall mean was 79.70 ± 12.66 µm. Compared to ROI1, Vybrant-CM-DiI labelled cells were located significantly (*p* = 0.0373) closer to the bone surface in ROI2 where the mean distance was 24.93 ± 4.85µm (Fig. 3C). In contrast in ROI2, the distance between individual tumour cells was significantly greater, 384.30 ± 129.80 µm in the growth plate area compared to the trabecular bone region, with mean values of 174.9 ± 26.07 µm (*p* = 0.0239) (Fig. 3D). In both ROIs, the bone-seeking cell-line MDA-MB-231-GFP-IV homed significantly closer to the nearest bone surface than to the nearest tumour cell (*p* = 0.0015 in ROI1 and *p* = 0.0214 in ROI2, Fig. 3E and 3F). Taken together, our data show that DTCs home to niches in close proximity to the nearest bone surface and that tumour cells are not found to cluster together.

### 3.3 MDA-MB-231-GFP-IV cells homing to bone is not affected by the route of administration

**Fig. 4:**
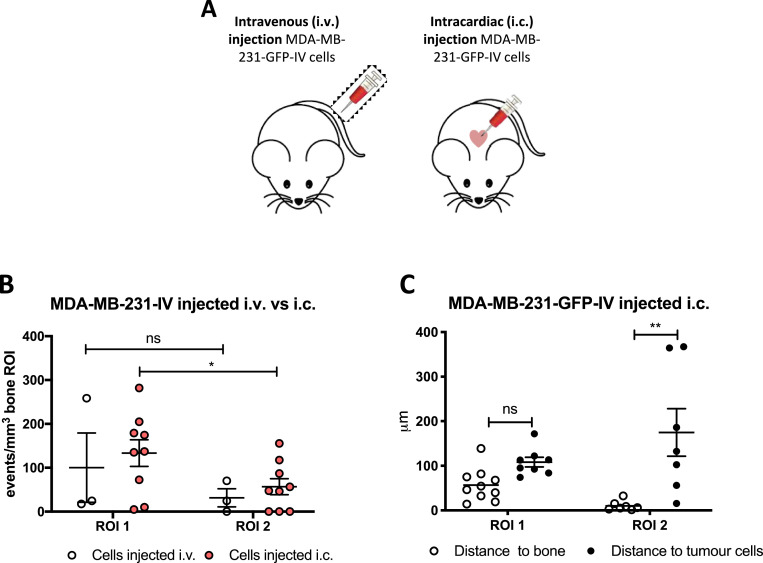
Comparison between different routes of tumour cell injection. (A) Experimental outline of the *in vivo* study. 12-week old female BALB/c nude mice were injected either i.v. (*n* = 1) or i.c. with 1 × 10^5^ MDA-MB-231-GFP-IV cells labelled with Vybrant-DiD (*n* = 5) to compare the pattern of homing using different routes of injection. (B) Graph representing the direct comparison between the homing of MDA-MB-231-GFP-IV cells after i.v and i.c injection. Comparison between the distance to the nearest bone surface and to the closest tumour cells after i.c. injection is show in (C). *p ≤ 0.05 and **p ≤ 0.01 student's *t*-test and two-way ANOVA and Tukey *post-hoc* test.

Due to their characteristic bone tropism, MDA-MB-231-GFP-IV cells home to the long bones following tail vein injection [29]. In contrast, most xenograft models of bone metastasis involve administration of tumour cells via the intracardiac route, allowing the cells to reach the bone through the arterial circulation. To determine whether the route of injection modifies the initial stages of breast cancer cell bone colonisation, 12-week old BALB/c nude mice were injected with MDA-MB-231-GFP-IV Vybrant-DiD labelled cells either i.v. (*n* = 1, to confirm the pattern described above) or i.c. (*n* = 5) and after 5 days long bones were collected and tumour cells were visualized in the bone microenvironment and quantified using two-photon microscopy (Fig. 4).

When injected via the tail vein, MDA-MB-231-GFP-IV cells displayed the same pattern of homing as described above (ROI1>ROI2), but did not show significant differences between events recorded in the trabecular region of the bone compared to the growth plate area (*p* = 0.4480) (Fig. 4B). This was probably due to the limited number of samples available for analysis.

When injected via the intracardiac route, the majority of MDA-MB-231-GFP-IV cells still located in ROI1 compared to ROI2 (*p* = 0.0464) (Fig. 4B). Moreover, the tumour cells colonised areas in close proximity to the bone surface with a mean value of 56.99±11.55µm in ROI1 and 10.30±4.04µm in ROI2 (*p* = 0.0053). As was observed following i.v. injection, the distance to the nearest other tumour cell was higher than that to the nearest bone surface, mean value of 253.50±110.40µm in ROI1 and 174.90±53.36µm in ROI2 (*p* = 0.1225 and *p* = 0.0096 in ROI1 and ROI2 respectively) (Fig. 4C).

Taken together, these results show tumour cells capable of metastatic outgrowth home to trabecular bone irrespective of their route of injection and confirm that triple negative breast cancer cells generally seed in the trabecular region of the metaphysis in close proximity to the bone surface.

### 3.4 ER+ve breast cancer cell-lines exhibit the same pattern of seeding as triple negative cell-lines

**Fig. 5:**
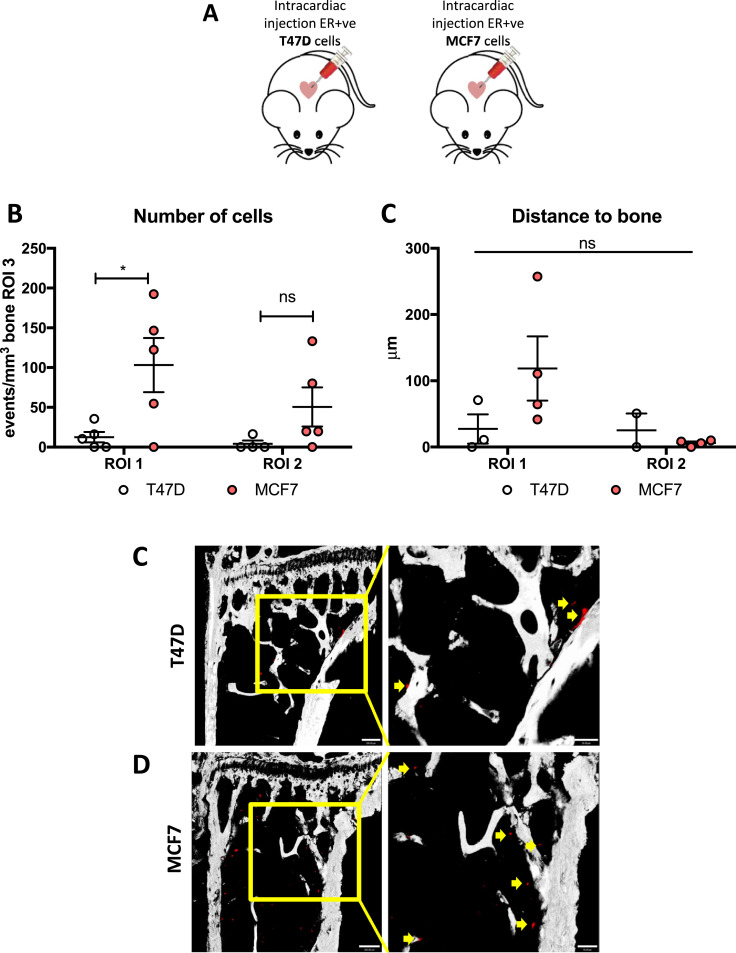
Homing of ER+ve cell lines. (A) Experimental outline of the *in vivo* study. 12-week old female BALB/c nude mice were injected on day 1 with 1 × 10^5^ T47D or MCF7 Vybrant-DiD labelled cells i.c. (*n* = 5/group) to compare the homing pattern of ER+ve cell-lines with the triple negative MDA-MB-231. Five days later, animals were culled and long bones collected for *ex-vivo* two-photon analysis. (B) Graph representing the direct comparison between the homing of T47D cells *vs* MCF7 cells (*n* = 5 bones/group analysed). (C) Comparison between the distances to the nearest bone surface of the two cell-lines injected. *p ≤ 0.05 two-way ANOVA and Tukey post-hoc test. Examples of two-photon scan showing the proximity to the bone surface of T47D (D) and MCF7 (E), position of the cells is highlighted by the yellow arrows and scale bar are 100 µm (right panel) and 50 µm (left panel). (For interpretation of the references to color in this figure legend, the reader is referred to the web version of this article.)

The majority of breast cancers are ER +ve, and bone metastasis is more commonly seen in patients with ER+ve tumours. In contrast, most *in vivo* models of bone metastasis use triple negative breast cancer cell-lines (e.g. MDA-MB-231). We next investigated whether ER+ve breast cancer cells colonised bone in the same way as ER-ve breast cancer cells. For these experiments, the ER+ve cell lines T47D and MCF-7 were labelled with Vybrant-DiD and injected into 12-week old mice via the intra cardiac route (Fig. 5A).

Similar to the triple negative breast cancer cell-lines, the ER+ve cell-lines also displayed a trend towards homing to the trabecular region of the bone compared to the area adjacent to the growth plate, although the *p*-value did not reach statistical significance (*p* = 0.3481 for T47D and *p* = 0.2457 for MCF7 cell-line, Fig. 5B-D). Interestingly, the number of T47D cells reaching the bone microenvironment (ROI1) was lower than for the MCF7 cells, (*p* = 0.0472) (Fig. 5B).

MCF7 cells appeared to home slightly further from the bone surface in ROI1 compared to all the other cell-lines analysed, with a mean value of 118.50 ± 48.44 µm (42–257 µm) while in ROI2 the mean distance of 6.09 ± 2.21 µm (0–10 µm) is comparable to both the triple negative cell-line and the ER+ve clone T47D (Fig. 5C).

Of all the cell-lines investigated, T47D cells were in closest proximity to the bone surface, with a mean distance of 27 µm (0–71 µm) to the nearest bone surface in ROI1, suggestive of breast cancer cell sub-type specific differences in niche location (Fig. 5C). These results confirm that both ER+ve and ER-ve breast cancer cells colonise the same region of the bone microenvironment.

### 3.5 Breast cancer cells colonise the same areas of bone in both young and mature mice

**Fig. 6:**
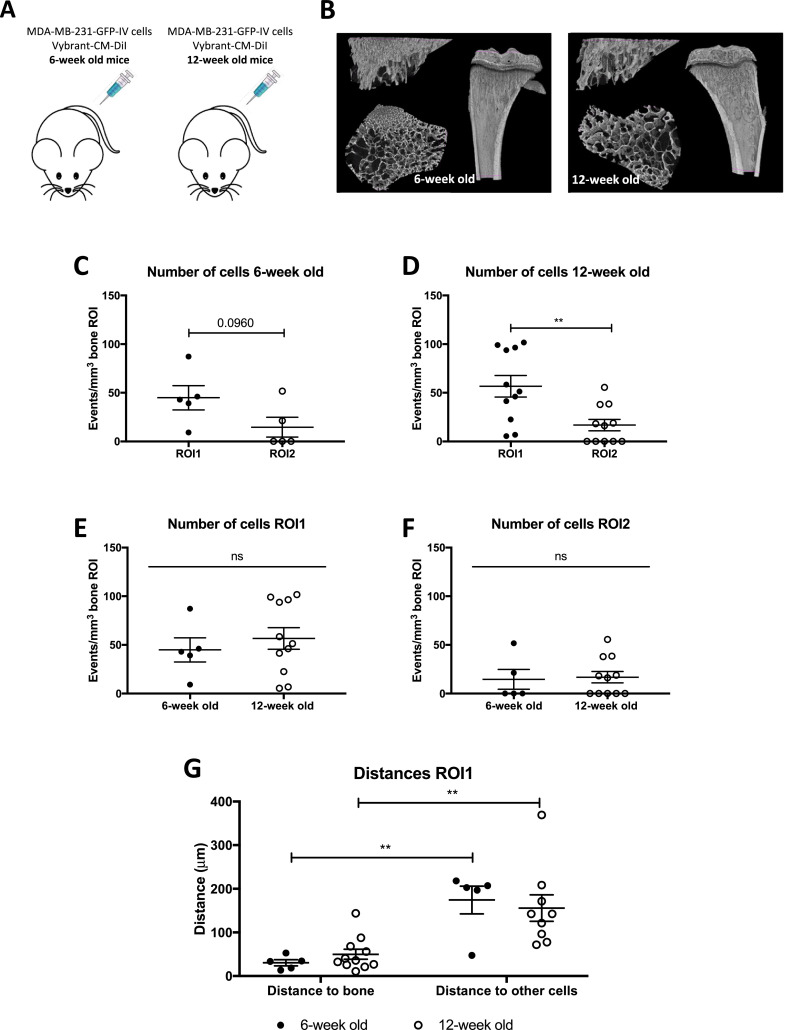
Homing of MDA-MB-231-GFP-IV cells in young and mature mice. (A) Experimental outline of the *in vivo* study. 6- and 12-week old female BALB/c nude mice (*n* = 8/group) were injected on day 1 with 1 × 10^5^ CM-DiI labelled MDA-MB-231-GFP-IV cells i.v. Five days later, animals were culled and long bones collected for *ex-vivo* two-photon analysis. (B) The number of MDA-MB-231-GFP-IV cells detected in 6- and 12-week old mice 5 days after tumour cell i.v. injection (*n* = 8/group), graph shows mean ± SEM. (C) Distances of the tumour cells to the nearest bone surface and to the closest breast cancer cells. Two-way ANOVA and Tukey's *post-hoc* test.

We have previously shown that young mice (<6-week old) develop bone metastases with higher frequency when compared to mature (>12-week old) mice following tumour cell administration, and that this is associated with differences in bone turnover which is higher in young compared to mature animals [30], [31]. To establish if this is due to a difference in the number of breast cancer cells reaching the bone microenvironment in mice of different ages, 1 × 10^5^ MDA-MB-231-GFP-IV cells labelled with Vybrant-CM-DiI were injected i.v. into 6- and 12-week old BALB/c nude mice (Fig. 6). Five days after injection the animals were culled and long bones were collected for two-photon microscopy (femora) and for micro-computed tomography (μCT) and fluorescent immunohistochemistry (tibiae). Analyses were performed in tumour-cell bearing bones only (*n* = 5 for 6-week old and *n* = 11 for 12-week old mice). There was a marked difference in the bone architecture of the metaphysis between young and mature animals as illustrated by representative images in Fig. 6B. The µCT analysis revealed that although there was no difference in the trabecular bone volume between the two different age groups (*p* = 0.1023), there was a significant difference in both the number and thickness of the trabeculae. The trabeculae of young animals were significantly thinner (*p* < 0.0001) and their number was significantly higher (*p* = 0.0034) when compared with mature animals, demonstrating that the area of bone containing/comprising the metastatic niche is altered with age (Supplementary Fig. 1).

The preferential homing pattern of breast cancer cells was maintained, with fewer DTCs detected in ROI2 compared to ROI1, although this did not reach statistical significance in the young animals (*p* = 0.0960 for 6-week old and *p* = 0.0047 for 12-week old animals) (Fig. 6C and D). Moreover, despite the identified differences in bone architecture, there were no significant differences in the average number of breast cancer cells located in ROI1 (44.91 ± 12.46 in 6-week old and 56.64 ± 11.05 in 12-week old, *p* = 0.5372) and ROI2 (14.60 ± 10.15 in 6-week old and 16.83 ± 5.91 in 12-week old, *p* = 0.8430) between the different age groups (Fig. 6E and F).

Irrespective of age, breast cancer cells located close to the bone surface in the trabecular region, with a mean value of 30.49 ± 6.92 µm (13–52 µm range) in 6-week old and 49.97 ± 11.55 µm (10–143 µm range) in 12-week old mice. Breast cancer cells preferentially home significantly closer to the bone surface than to other tumour cells (174.40 ± 31.94 µm, *p* *=* 0.0064 in 6-week old and 155.80 ± 30.49µm, *p* *=* 0.0047 in 12-week old) (Fig. 6G). Distances were not measured in ROI2 due to the low number of bones in which tumour cells were detected in this region.

Taken together, these results confirm that the pattern of breast cancer bone colonisation is independent of animal age, with a significantly higher number of events, closer proximity to the nearest bone surface and a significantly higher distance to the nearest tumour cell, in ROI1 compared to ROI2.

### 3.6 Modification of the HSCs niche changes the homing of breast cancer cells

**Fig. 7:**
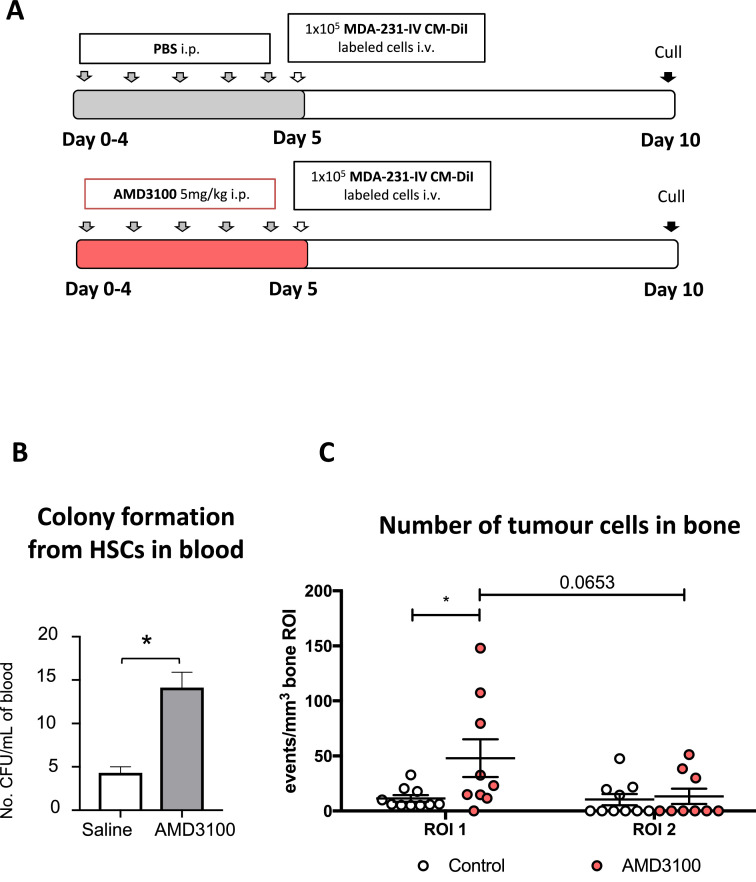
Modification of the HSCs niche – effect on tumour cell homing. (A) Experimental outline of the *in vivo* study. 12-week old female BALB/c nude mice were injected daily for 5 days with PBS or AMD3100 (5 mg/kg i.p.) (*n* = 5/group). On day 5, animals were injected with 1 × 10^5^ Vybrant-DiD labelled MDA-MB-231-GFP-IV cells i.v. and culled on day 10, tibias were collected for two-photon microscopy. (B) *Ex vivo* colony formation by HSC/PCs present in the peripheral blood of 12-week old female BALB/c-Nude mice treated daily for five days with AMD3100 (5 mg/Kg, i.p.) or saline. Graph shows HSC/PCs colony numbers after 11 days of culture *ex vivo*. (*p<0.05 Mann-Whitney *U test; n* = 4/group). (C) Graph representing the homing of MDA-MB-231-GFP-IV cells after treatment with the CXCR4 antagonist AMD3100 (*n* = 9 bones analysed) or with PBS (*n* = 10 bones analysed). Significantly greater number of tumour cells was detected in ROI1 after the mobilization of the HSCs cells outside the niche. *p ≤ 0.05 two-way ANOVA and Tukey's *post-hoc* test.

It has been suggested that the metastatic niche broadly overlaps with the hematopoietic stem cell niche in the trabecular region of the metaphysis of long bones. Shiozawa et al. elegantly demonstrated that prostate cancer cells and HSCs locate within the same niche [20], [21] using a CXCR4 antagonist (AMD3100) to mobilize the HSCs, but it is not known whether this is also the case for breast cancer cells. We therefore used a similar approach to investigate whether mobilisation of HSCs from the bone niche into the circulation would increase the number of sites available for tumour cells to colonise, as outlined in Fig. 7A. To allow direct comparison with the Shiozawa study, the same dose of AMD3100 (5 mg/kg i.p) shown in their study to mobilise HSCs [20] was injected daily for 5 days in 12-week old mice. As shown in Fig. 7B, after 11 days of culture *ex vivo* there was a significant increase in colony numbers generated from HSC/and other progenitor cells (PCs) isolated from blood samples of AMD3100-treated mice compared to control, demonstrating successful mobilization of HSCs/PCs. Next, Vybrant-CM-DiI labelled MDA-MB-231-GFP-IV cells were injected into the tail vein of animals that had received 5 days of AMD3100 or control treatment. Animals were culled 5 days following tumour cell injection and the number and location of tumour cells in the bone marrow was compared between control and AMD3100 treated animals using two-photon microscopy.

There was a significantly greater number of tumour cells colonizing the trabecular region (ROI1) of the bone in AMD3100 treated mice compared to control (*p* *=* 0.0409). In contrast, no difference in tumour cell number was observed in the growth plate area of the bone (*p* = 0.7303), which is usually less colonised and does not contain HSCs niches (Fig. 7B). Alteration of the HSCs population resulted in a modification of the number of tumour cells locating to bone niches, supporting that breast cancer cells home to HSC niches when arriving in bone.

### 3.7 DTC in bone marrow locate adjacent to components of the perivascular niche

**Fig. 8:**
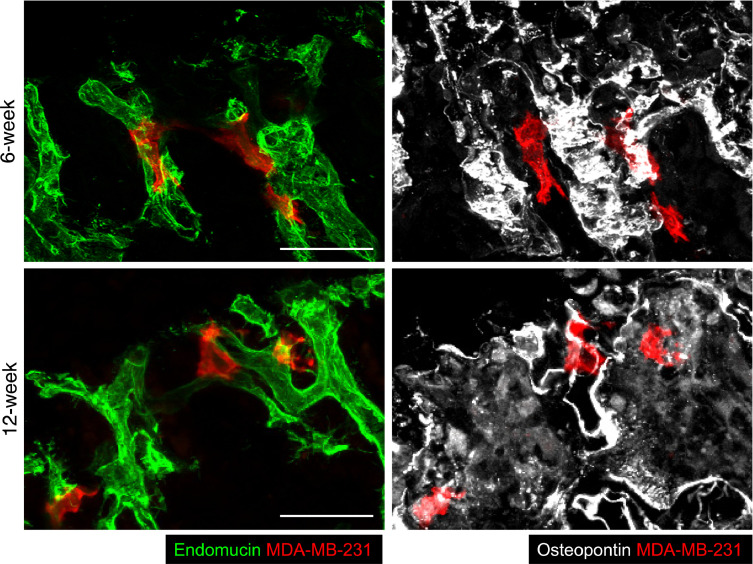
Disseminated breast cancer cells reside in perivascular locations within mouse long bones. Immunofluorescent labelling of tibial cryosections from young and mature mice containing human MDA-MB-231 breast cancer cells with antibodies against the vascular marker Endomucin, the bone marker Osteopontin and two human antigens expressed by MDA-MB-231 cells. The samples were prepared 6 days after intra-cardiac injection of tumour cells (scale bar = 50 µm). (For interpretation of the references to color in this figure, the reader is referred to the web version of this article.)

The exact composition of the metastatic niche has not yet been elucidated, but tumour cells are reported to locate in regions of bone where the HSCs, endosteal and perivascular niches overlap [14], [15], [16], [17]. The perivascular niche in particular has been suggested to influence the fate of DTC by modulation of the expression of Thrombospondin-1 (supporting dormancy) and TGF-β1 and periostin (promoting tumour growth). To investigate the spatial relationship between breast cancer cells, endosteal surfaces and bone microvessels, 30 µm thick tibial sections of long bones, isolated from 6-week and 12-week old animals 6 days following tumour cell injection, were stained with antibodies against endomucin (a pan-endothelial marker to visualise the entire capillary network), the bone marker osteopontin and human CD29 and CD59 (markers expressed on the human tumour cells). As shown in Fig. 8, the tumour cells (red) were found outside vessels but immediately adjacent to endothelial cells (green, left hand panels), closely associated with bone (white, right hand panels). Our data are in agreement with tumour cells locating to in the perivascular niche at this time point. Future studies will include quantification of DTCs in relation to the different vessel subtypes.

## 4 Discussion

Bone metastasis remains a major clinical challenge in advanced breast cancer, and is frequently associated with pain, loss of mobility and in severe cases pathological fractures. Current standard therapies for established bone metastasis are anti-resorptive agents that act to slow disease progression and improve quality of life but without improving survival [5]. Elucidating the role of the microenvironment in supporting tumour development and progression is key to improving cancer therapies, as targeting the tumour cells alone is not sufficient to eradicate disease in the majority of cases [33] In particular, the use of bone-targeted agents to modify bone marrow niches from a supportive to a ‘hostile’ environment has been widely investigated, including the evaluation of anti-resorptive bisphosphonates in large adjuvant trials in breast cancer [34]. However, there is currently no effective therapy for general prevention of bone metastasis, thus to develop additional therapeutic agents with enhanced efficacy, we need a more comprehensive understanding of the key interactions that facilitate the early stages of tumour cell colonisation and survival in bone. Whilst still impossible in humans, the use of *in vivo* model systems allows the detection of individual (labelled) tumour cells *ex vivo* and mapping of their location and proximity to different cell types in bone at different time point. In this study, we have used a combination of murine bone metastasis models and advanced imaging to determine the early stages of tumour cell colonisation of bone, comparing ER+ve and ER-ve cells, different injection routes and recipient ages, in addition to evaluating how pharmacological alteration of the microenvironment modifies subsequent tumour cell homing.

Despite the large number of breast cancer cells that were successfully injected into the circulation of our animal models, only a small portion colonized the long bones of immunocompromised mice. In the bone metastasis models used in this study, the majority of tumour cells are eliminated by day 5 due to a combination of factors; cell death in the circulation, inability to locate an appropriate niche and/or elimination by innate immune cells. This observation is in agreement with numerous studies using xenograft models of bone metastasis showing that only a limited number of tumour colonies (generally 1–5) develop in individual bones following injection of ∼10^5^ tumour cells. As in human disease, the data from *in vivo* models (including the current study) demonstrate that simply arriving in bone is not sufficient to cause the secondary tumour [3], [5], [6], [35]. Malignant cells must be able to interact with the microenvironment in order to receive and respond to signals that control their survival and proliferation [36], [37]. We have carried out the first detailed mapping of the tumour cells to establish whether they locate close to the endosteal niche (nearest bone surface) or whether they locate close to other tumour cells. Our data show that individual tumour cells do not locate closely together, suggesting that cells that have successfully found a suitable niche do not signal to other tumour cells to home to the same space. This could suggest that there is a limited number of available niches that could be saturated, meaning that there is competition for the metastatic niches in bone, although this needs to be further investigated. In all cases, tumour cells were found closer to the nearest bone surface than to other tumour cells, suggesting that cells preferentially home to endosteal niches.

Most studies using murine bone metastasis models are carried out in young (∼6-week old) animals with high levels of bone remodelling, in order to maximise tumour development and allow measurement of therapeutic effects. In contrast, we have shown that when tumour cells are injected into animals with a more mature skeleton (>12-week old), only around 20% of the mice will develop overt tumour growth in bone. In the remaining 80%, the tumour cells home to bone but remain dormant in the absence of accelerated bone turnover [30]. Several studies, including from our own laboratory, have shown that subsequent alteration of the bone microenvironment to accelerate bone turnover such as OVX [38] or castration [39] triggers the growth of the disseminated tumour cells to form bone metastases. These studies highlight the major differences between tumour growth in young compared to mature mice, but is this due to the ageing bone microenvironment having reduced capacity to support tumour growth, or could it also be due to increased numbers of tumour cells reaching bone in the young animals? We answered this question by injecting young and mature animals with the same number of breast cancer cells and quantifying their abundance and location 5 days later. There were no significant differences in the early steps of the breast cancer dissemination to bone between young and mature animals, neither in the number of cells homing to bone nor in their location within the bone microenvironment. These results support the hypothesis that the differences in subsequent tumour outgrowth are due to tumour cells receiving signals from the microenvironment, rather than to differences in how and where the tumour cells reach the metastatic niches in young and mature animals. These results are in agreement with our earlier study using DiD-labelled MDA-MB-231 or PC3 prostate cancer cells injected in 6- and 16-week old mice via the intra-cardiac route, resulting in comparable numbers of tumour cells located in the long bones of animals of both ages 7 days after injection [30]. Taken together, these studies provide evidence that tumour cells locate to bone marrow niches with the same frequency in young and mature animals, despite the considerable differences between the bone microenvironments.

The trabecular areas of mouse long bones contain a complex and extensive microvascular network, recently reported to consist of distinct vessel subtypes (termed H and L vessels) with potential differential function [22]. H vessels located in the metaphysis form a novel, structurally distinct capillary subtype, shown to mediate microvascular growth and maintenance of perivascular osteoprogenitors, thereby coupling angiogenesis and osteogenesis. We found that the majority of tumour cells were located in the region of trabecular bone (RO1) that contains H vessels, suggesting that there could be interactions between disseminated tumour cells and this specific vessel type, although this warrants further investigation. As two-photon microscopy is not suited to visualise multiple cell types in the same sample, we used confocal microscopy to determine whether MDA-MB-231 breast cancer cells located to sites in close proximity to endothelial cells. We found that 6 days after injection, breast cancer cells were situated in perivascular sites in the trabecular areas of bone, consistent with the hypothesis that the endosteal and perivascular niche areas broadly overlap in this region. Studies using *ex-vivo* models have reported that the microvasculature regulates the quiescent state of breast cancer cells, and that expression of thrombospondin (TSP-1) by the stable microvasculature supports dormancy whereas pro-tumourigenic molecules (like periostin and TGF-b1) produced during vessel sprouting stimulates tumour cell proliferation [26]. We were unable to determine whether tumour cells were more closely associated with osteoblastic cells compared to cells of the microvasculature, both being highly abundant in the area of bone where tumour cells wer located. We could therefore not establish whether either of these cell types are likely to provide more dominant regulation of tumour cell dormancy/proliferation in this model system, as soluble factors released locally would reach the tumour cells regardless of whether the source was the microvasculature or the bone cells. However, as shown in Fig. 8, Fig. 6 days after injection, tumour cells were found located immediately adjacent to endothelial cells and appeared to wrap around the outer surface of the vessels, supporting a potential direct interaction between tumour cells and endothelial cells.

A major criticism of murine breast cancer bone metastasis models is the almost exclusive use of ER-ve tumour cells, despite the majority of patients developing bone metastases having ER+ve disease [40]. The lack of representative ER+ve models is mainly due to the need for animals to receive oestrogen supplementation for tumours to develop, although ER+ve mouse mammary tumour cell lines have been developed that form colonies in bone independently of oestrogen in immunocompetent mice [41]. We have previously shown that oestrogen has a major impact on the bone microenvironment, its potent bone anabolic effects altering the biology of the niche, precluding studies of bone metastasis [42]. However, here we hypothesised that the early homing steps and survival of ER+ve tumour cells for the first few days in bone are hormone-independent and can be studied in the absence of oestrogen supplementation. Comparing the homing of different breast cancer cell lines to bone, we found that the preferred location of ER+ve tumour cells in bone was comparable to that of ER-ve cells, although some minor differences between the different cell lines were observed. Our data provide valuable new information to show that the early steps of tumour cell dissemination in bone is not dependent on ER status of the tumour cells or require oestrogen supplementation to be successful, opening the field for further studies of the mechanisms regulating initiation of bone metastasis in ER+ve breast cancer.

Breast cancer cells home to the same areas of bone that harbour the HSC niche, and using prostate cancer models Shiozawa and colleagues demonstrated that mobilisation HSCs caused by administration of the CXCR4 antagonist AMD3100 *in vivo* increased the number of prostate cancer able to seed in the bone [20], [21]. They concluded that prostate cancer cells located to HSC niches, however whether this also applies to breast cancer cells has, to our knowledge, not been investigated. As the MDA-MB-231-IV cells used in our study do not express CXCR4, possibly due to the cycles of *in vivo* selection to make them home to bone, AMD3100 could not be used to mobilise them from the bone marrow into the circulation. We therefore used two-photon microscopy to establish mobilisation of HSCs prior to tumour cell injection resulted in alterations in the location and/or number of breast cancer cells within bone. We provide the first demonstration that, in agreement with reports from prostate cancer models, modification of the HSCs population resulted in alteration of the overall number of ER-ve breast cancer cells detected in the bone microenvironment. Our data support that breast cancer cells locate to HSCs niches within bone and that mobilisation of HSCs appears to free up space for tumour cells to colonise, thereby potentially increasing the risk of developing bone metastases. Our data provide strong evidence to support that breast cancer cells locate to similar locations within the long bones irrespective of their ER status, hence likely colonise the same niches, although further studies would be needed to conclusively show that ER+ve breast cancer cells home to HSC niches. It is possible that administration of AMD3100 could influence bone-resident cell types other than HSC/progenitor cells. However, significant effects on bone turnover have only been reported after prolonged treatment with AMD3100. Im et al. reported that AMD3100 treatment (5 mg/kg/day) for 21 consecutive days induced release of SDF-1, preventing ovariectomy-induced bone loss in female C57BL/6 mice by decreasing the number of osteoclasts differentiated from BM-derived precursors [43]. In contrast, short term administration of 5 mg/kg/day AMD3100 to C57BL/6 mice for 3 days is reported not to affect bone mineral density *in vivo* [44]. Using a prostate cancer model, Zalucha et al. reported that male SCID mice administered AMD3100 (5 mg/kg daily for 5 days) had increased number of osteoclasts compared to control accompanied by a trend towards increased seeding of subsequently injected prostate cancer cells in the bone marrow [45].

Taken together, this study provides valuable new information about how *in vivo* models of breast cancer bone metastasis can be combined with advanced imaging to establish the early steps of tumour cell colonisation of bone. We demonstrate that both ER+ve and ER-ve breast tumour cells homing to long bones is microenvironment dependent, with cells locating to highly vascularised, trabecular areas in both young and mature animals. We also show that the homing pattern is unaffected by the cellular dye used to label the tumour cells and by the route of administration, supporting that studies using different methodologies could be broadly comparable. Finally, we provide evidence that breast cancer cells home to the HSC niche, supporting that the HSC, perivascular, endosteal and metastatic niche are overlapping in this model system.

## 5 Conclusions

Our findings demonstrate that in mouse models of bone metastasis, breast cancer cells home to specific bone sites irrespective of their ER status, the age of the recipient animal or the injection route, supporting that tumour cells home to niche areas where the bone microenvironment is supportive of their colonisation and survival. Tumour cells locate closer to the nearest bone surface than to other tumour cells, suggesting that the environment, not the presence of other cancer cells, supports tumour cell homing by providing suitable niches. Mobilisation of HSC resulted in increased numbers of tumour cells homing to bone, supporting that the metastatic niche and the HSC niche are overlapping.

## Declarations

 

## Funding

This study was supported by Breast Cancer Now, UK (2014NovPhD401); Cancer Research UK (C8525/A21082) and the Marie Curie ITN “Bone Net”.

## Appendix Supplementary materials

Image, application 1

Image, application 2
