# Supplementary material for: The bone metastasis niche in breast cancer-potential overlap with the haematopoietic stem cell niche in vivo
Source: J Bone Oncol. 2019 Jun 7;17:100244. doi: 10.1016/j.jbo.2019.100244 (PMC6582079; doi:10.1016/j.jbo.2019.100244)
Supplement: Supplementary file 2 [file mmc2.docx]

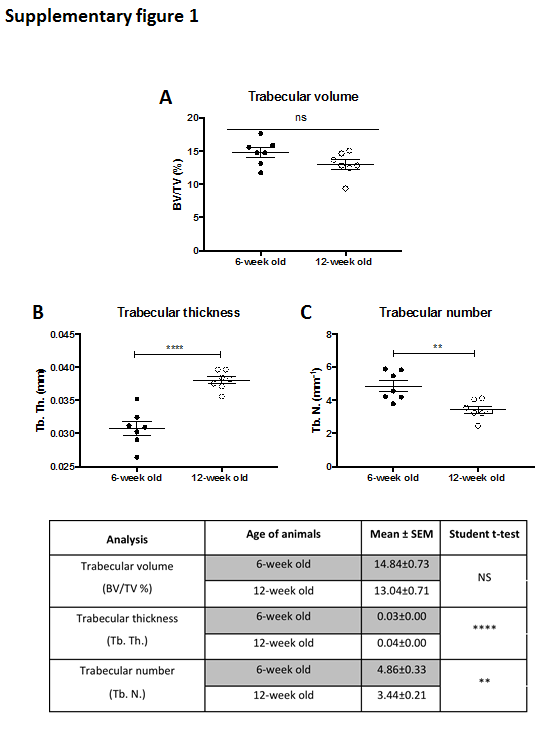


**Supplementary Figure 1. Quantification of trabecular bone volume, thickness and number in young and mature mice.** A) Quantification of trabecular bone volume (BV/TV), (B) trabecular thickness and (C) number of tibiae of 6- and 12-week old animals (n=7/group). (D) Summary table of µCT analyses. Student t-test, **p<0.01, ***p<0.0001, ns =not significant.
